# Intracerebral Hemorrhage Caused by the Rupture of a Traumatic Pseudoaneurysm in the Middle Meningeal Artery

**DOI:** 10.3390/jcm12237337

**Published:** 2023-11-27

**Authors:** Myoung Soo Kim, Younghwan Kim

**Affiliations:** 1 Department of Neurosurgery, Trauma Center, National Medical Center, Seoul 04564, Republic of Korea; 2 Department of Surgery, Trauma Center, National Medical Center, Seoul 04564, Republic of Korea; galgunbam2@nmc.or.kr

**Keywords:** meningeal artery, aneurysm, trauma, cerebral hemorrhage

## Abstract

Hematomas caused by the rupture of a pseudoaneurysm in the middle meningeal artery (MMA) after trauma usually present as epidural hematomas. Intracerebral hemorrhage (ICH) is extremely rare. We reviewed ICH due to the rupture of MMA pseudoaneurysms. We found that in cases of acute ICH, a pseudoaneurysm was attached to the outer surface of the dura mater and associated with dura tear. In patients with acute ICH, the intraoperative rupture of a pseudoaneurysm developed just after bone flap removal. In cases of delayed ICH, pseudoaneurysms adhered to the inner surface of the dura mater. In patients with delayed ICH, the intraoperative rupture of a pseudoaneurysm developed during dura opening and hematoma removal. In situations of dura tear after trauma, the rupture of pseudoaneurysms might lead to ICH via a dura tear. Pseudoaneurysms that develop in the MMA after trauma may exert pressure and result in the thinning of the dura mater. In this case, pseudoaneurysms will adhere to the inner surface of the dura mater after several days or weeks. ICH might develop through both acute and delayed mechanisms following the development of pseudoaneurysms in the MMA. Clinicians should pay attention to the timing of such ruptures during operations for both acute and delayed ICH.

## 1. Introduction

Pseudoaneurysm in the middle meningeal artery (MMA) after trauma is an uncommon entity. Pseudoaneurysms develop from the rupture of all three layers of the arterial wall. True aneurysms arise from partial damage to the arterial wall with an intact adventitia. The most common clinical manifestation of a traumatic pseudoaneurysm in the MMA is an epidural hematoma (EDH). In 1957, Schulze, reported the first case of traumatic pseudoaneurysm in the MMA presenting with an EDH [1]. Intracerebral hemorrhage (ICH), or subdural hematoma (SDH), is an extremely rare presentation [2,3,4,5,6,7,8]. Pseudoaneurysms in the MMA carry a potential risk of rupture, producing neurological deterioration due to ICH after a 3 to 30 day interval [9]. The mechanism of acute and delayed ICH development due to the rupture of MMA pseudoaneurysms is not well understood. Some investigators have proposed that the delayed growing of a pseudoaneurysm in the MMA and subsequent thinning of the dura mater might lead to ICH or an SDH [10,11]. Moon et al. suggested that the rupture of a pseudoaneurysm in the MMA in contact with the dura tear due to trauma could cause acute ICH via this dura tear [6]. Inappropriate craniotomy might result in a failure to identify pseudoaneurysms, and the intraoperative rupture of a pseudoaneurysm might also cause a difficult operation [4,6]. Here, we discuss the pathophysiological mechanisms of acute and delayed ICH due to the rupture of a traumatic MMA pseudoaneurysm. We also discuss the risks of surgical treatment, especially in regard to the extent of craniotomy and the timing of the intraoperative rupture of a pseudoaneurysm.

## 2. Materials and Method

### 2.1. Search Strategy

A search for eligible studies was conducted in the PubMed database without restrictions on the language or publication time. The search strategy combined the terms “middle meningeal artery”, “trauma”, “injury”, “cerebral hemorrhage”, and “subdural hemorrhage” using multiple forms of medical subject heading (MESH) terms and text words. The reference lists of the eligible studies and internet sources (Google Scholar, https:scholar.google.com, accessed on 1 August 2023) were evaluated by a manual search. The references of the studies were evaluated to determine additional eligible studies that were not included during the other search. The abstracts of conference proceedings were not evaluated because of the limited information on their operative findings. 

### 2.2. Study Selection

We included articles describing ICH due to an MMA pseudoaneurysm developing after trauma. Potentially eligible articles were selected for full-text evaluations. The exclusion criteria were studies performing endovascular treatment for MMA pseudoaneurysms, articles describing incidental MMA pseudoaneurysms, and cases presenting as EDHs by the rupture of an MMA pseudoaneurysm. Articles lacking clinical data (especially detailed operative descriptions), pseudoaneurysms in the MMA associated with arteriovenous fistula in the dura mater, and no operative treatment for MMA pseudoaneurysms were also excluded. 

### 2.3. Data Extraction

For each study matching the inclusion criteria, the authors extracted the following data: age and sex of the patients, type of trauma, location of ICH, acute or delayed presentation of ICH after trauma, operation time from trauma, preoperative diagnostic modality of the pseudoaneurysm in the MMA, location of the pseudoaneurysm in the MMA (especially the relationship with the dura mater), intraoperative rupture of the pseudoaneurysm, treatment for the pseudoaneurysm. Cases with abnormal neurological conditions upon immediate examination after trauma were classified as acute ICH. Patients with delayed deterioration after trauma were classified as delayed ICH.

## 3. Results

### 3.1. Results of Search and Study Selection

We evaluated 40 articles for full-text reading and excluded 28 studies after the full-text evaluation. We excluded ten articles in which they performed endovascular treatment for MMA pseudoaneurysms [10,12,13,14,15,16,17,18,19,20], nine studies presenting EDHs by the rupture of MMA pseudoaneurysms [21,22,23,24,25,26,27,28,29], three presenting incidental MMA pseudoaneurysms [9,30,31], one article without operative treatment for MMA pseudoaneurysms [32], four articles describing pseudoaneurysms in the MMA associated with arteriovenous fistula in the dura mater [33,34,35,36], and one article describing non-traumatic aneurysm in the MMA [37]. 

Four articles describing acute ICH due to the rupture of a pseudoaneurysm in the MMA were included in this study. And eight studies with delayed ICH from the bleeding of a pseudoaneurysm in the MMA were evaluated in this study.

### 3.2. Extracted Data

The clinical data of four patients with acute ICH due to the rupture of a pseudoaneurysm in the MMA are presented in Table 1. The detailed clinical information in eight patients presented with delayed ICH from the bleeding of a pseudoaneurysm in the MMA are presented in Table 2. 

## 4. Mechanism of Acute and Delayed ICH Due to Rupture of MMA Pseudoaneurysm

At the point where the MMA enters the cranial cavity, its histologic appearance changes to that of a cerebral artery. Defects in the media are extremely common [41], and these structural characteristics may influence the susceptibility of meningeal arteries to aneurysmal formation after trauma [22]. The rupture of a pseudoaneurysm in the MMA after trauma commonly leads to an EDH. Salazar et al. reported a pulsating round dilation of the MMA attached to the outer surface of the dura mater [9]. Other researchers have described a pseudoaneurysm in the MMA after trauma leading to an EDH that was located on the outer dura layer [1,23,28,42,43]. 

Uncommonly, these may result in an SDH or ICH. However, the exact mechanism of ICH development after the rupture of an MMA pseudoaneurysm remains poorly understood.

### 4.1. Acute ICH Development

**Figure 1:**
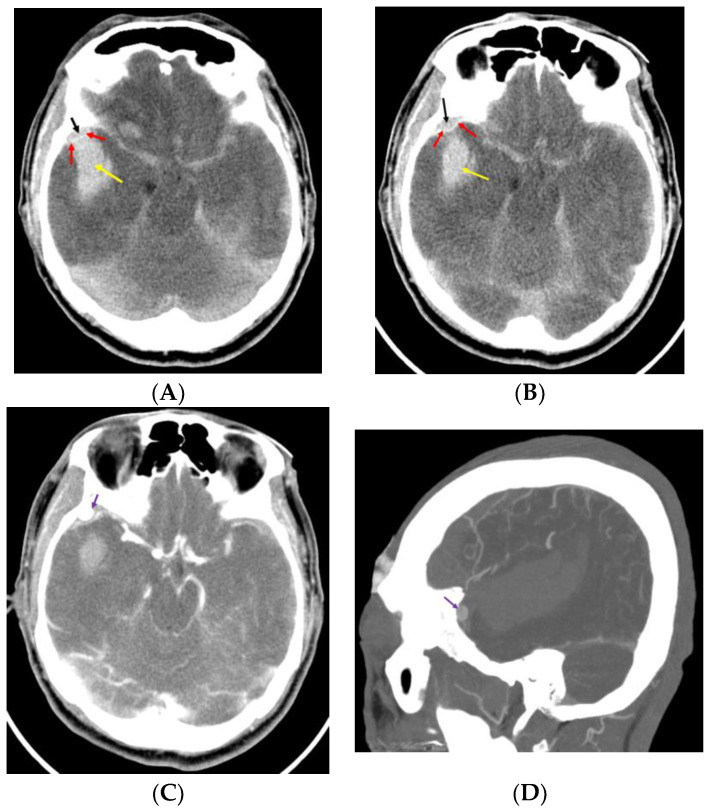
Preoperative computed tomography (CT) before (**A**,**B**) and after (**C**,**D**) contrast injection. Pre-contrast CTs demonstrate a small epidural hemorrhage (EDH) and intracerebral hemorrhage (ICH) (black arrow, EDH; red arrow, dura mater; yellow arrow, ICH). (**A**) ICH in contact with EDH. Post-contrast CTs (**C**,**D**) show a suspicious pseudoaneurysm located in the epidural space (violet arrow: suspicious pseudoaneurysm).

Rumbaugh et al. suggested that an SDH or ICH may result from the rupture of a pseudoaneurysm in the MMA, particularly if the dura has been torn; however, they did not present a case of dura tear resulting in ICH [44]. Kumar et al. and Moon et al. reported cases with an acute small EDH and ICH from the rupture of a pseudoaneurysm in the MMA after trauma [6,7] (Figure 1). 

**Figure 2:**
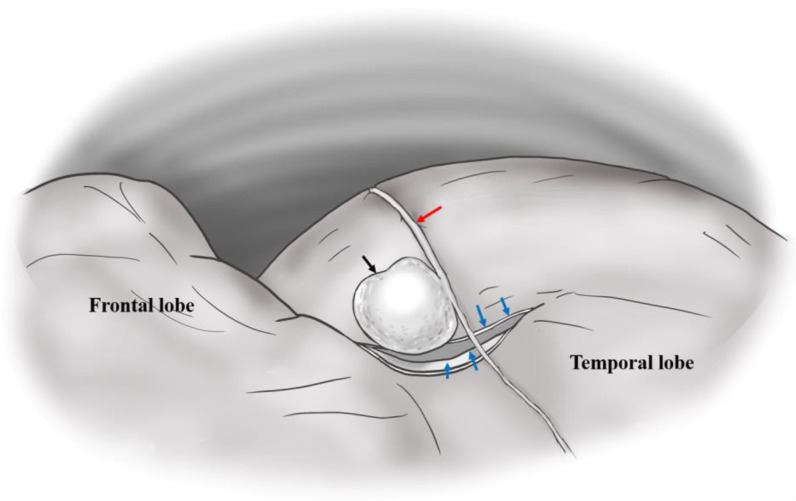
Dura tear associated with pseudoaneurysm in middle meningeal artery (MMA). (Blue arrow: dura tear, red arrow: MMA, black arrow: pseudoaneurysm).

In both reports, the authors mentioned that brisk epidural bleeding during bone flap removal and dura tear were detected. Moon et al. proposed that the rupture of a pseudoaneurysm in the MMA in contact with a dural defect could cause ICH via the dura tear [6]. In situations where there is a dura tear, the rupture of a pseudoaneurysm located in the epidural space produces an EDH at the initial stage of the rupture and subsequently results in ICH via the dura tear. Figures 1 and 3 in Moon et al.’s report [6] demonstrate that the rupture of a pseudoaneurysm in the MMA with a dura tear results in a small EDH and acute ICH. Figure 2 illustrates a dura tear associated with a pseudoaneurysm in the MMA.

### 4.2. Delayed ICH Development

**Figure 3:**
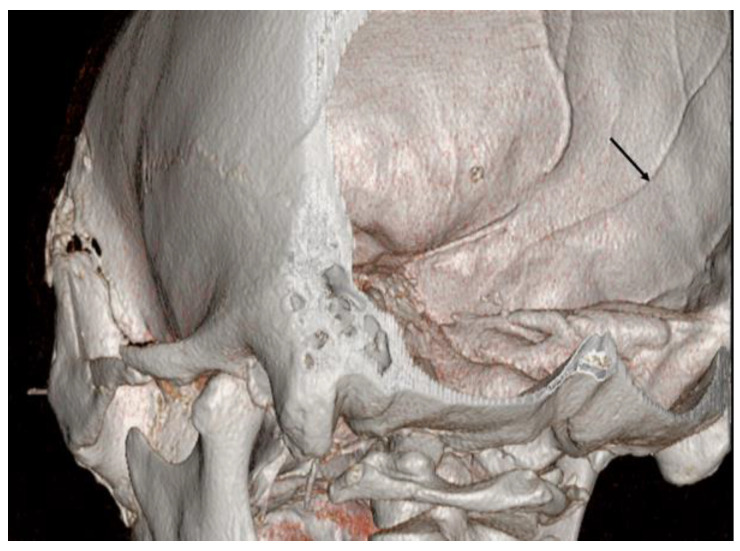
Reconstructed skull image shows vascular marking (arrow) due to the middle meningeal artery in the middle cranial fossa.

The dura mater has three distinct layers. The outer layer is the thinnest layer, at 2 μm thick, whereas the inner layer is 8 μm thick and adhered to the arachnoid trabeculae. The middle vascular layer, within which the MMA runs, varies in thickness [10]. In other brain operations, the operator may observe the MMA on the outer surface of the dura but may not identify the groove of the MMA in the inner surface of the dura mater. In the middle cranial fossa, clinicians may observe vascular marking, as seen in Figure 3. 

Traumatic pseudoaneurysms in the MMA are associated with temporal bone fractures, which involve the MMA groove in about 70–90% of cases [15]. Various mechanisms for the development of a pseudoaneurysm in the MMA have been suggested, including an arterial tear by a skull fracture or by the separation of the dura mater from the temporal bone. When the pseudoaneurysm in the MMA is not associated with a skull fracture or when they occur at locations distant from the fracture, traction injury to the MMA has been proposed [2,8]. Most cases of pseudoaneurysms in the MMA are located on the outer surface of the dura mater. Therefore, acute or delayed EDHs from pseudoaneurysms in the MMA are the most prevalent presenting type [11]. 

**Figure 4:**
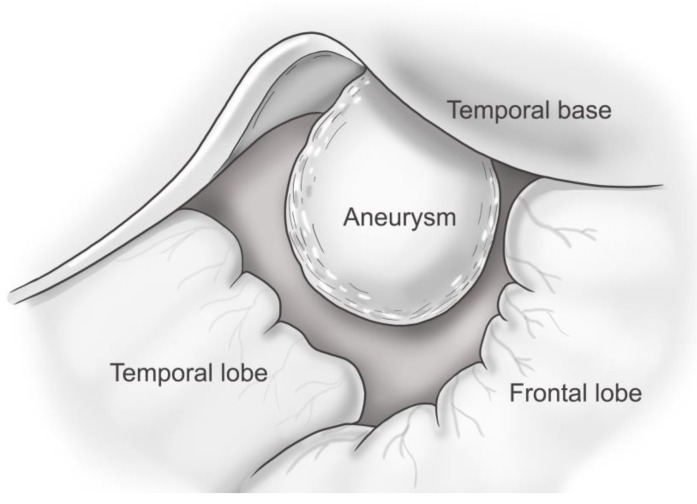
Pseudoaneurysm attached to inner surface of dura mater. This illustration was modified from Figure 2B in Aoki et al.’s report [4].

Regarding ICH development from a pseudoaneurysm in the MMA, Markwalder and Huber proposed that meningeal aneurysms attaching to the inner dural surface and the cerebral cortex are the consequences of traction on a small vessel bridging the meningeal and the cerebral vascular systems [45]. Some investigators have suggested that in cases of delayed ICH, the continuous pressure of the pseudoaneurysm thins the inner layer of the dura mater and consequently causes an SDH or ICH [10,22]. A pseudoaneurysm of the MMA in cases with delayed ICH has been shown to be attached to the inner surface of the dura mater [3,4,5,39,40] (Figure 4). It is thought that a pseudoaneurysm develops after a small tear in the MMA, which is sealed off by a clot, later recanalizing and forming a false lumen. A traumatic pseudoaneurysm in the MMA should be considered in patients who show delayed and abrupt neurological deterioration because of ICH after trauma [3]. In most reported cases of delayed ICH due to a pseudoaneurysm in the MMA, the usual delay between the trauma and abrupt neurological deterioration varies from 3 to 30 days [33]. Even so, at 11 months after trauma, delayed ICH from a pseudoaneurysm in the MMA could develop [3]. Sometimes, neither the families nor the patients recognize previous head trauma [38].

## 5. Preoperative Diagnosis of Pseudoaneurysm

### 5.1. Suspicion of Pseudoaneurysm

Although the incidence is rare, the concurrence of head trauma, a skull fracture located in the temporal area, and delayed neurological deterioration suggests the presence of a MMA pseudoaneurysm. About 70% of cases of pseudoaneurysms in the MMA after trauma are associated with a fracture crossing the MMA in the temporal region [15]. De Andrade et al. suggested that as many as 30% of patients with an EDH and a fracture crossing the groove of the MMA may have a pseudoaneurysm [21].

The first extracranial segment of the MMA is from its origin to its entry into the foramen spinosum. At the level of the foramen spinosum, the artery bends anteriorly and laterally to follow the temporal fossa. After its entry into the cranial cavity, the MMA follows a lateral course grooving the greater sphenoid wing. The intracranial segment of the MMA is divided into three portions. The first is the temporobasal segment, where the artery follows the temporal fossa and curves upward, becoming the second or temporopterional segment. After passing the pterional region, the artery enters the coronal segment, where it follows the coronal suture to the end of the region of the bregma [46].

### 5.2. Acute ICH following Rupture of Pseudoaneurysm

In preoperative diagnoses, acute ICH originating from an MMA pseudoaneurysm gives rise to the suspicion of a spontaneously ruptured cerebral aneurysm, which might have caused the patient’s trauma [47]. If acute ICH following trauma is in contact with the dura mater, clinicians should perform computed tomography (CT) angiography to detect a pseudoaneurysm. Paiva et al. and Marvin et al. reported that CT angiography detected pseudoaneurysms in the MMA [10,20]. Moon et al. and Kumar et al. identified the presence of a small EDH in contact with a large ICH due to the rupture of a pseudoaneurysm in the MMA [6,7]. From the mechanism of development of acute ICH following the rupture of an MMA pseudoaneurysm, we can expect that the intense contrast enhancement of the lesion in the epidural space adjacent to the EDH might indicate a pseudoaneurysm in the MMA.

### 5.3. Delayed ICH Due to Rupture of Pseudoaneurysm

Delayed ICH due to the bleeding of a pseudoaneurysm in the MMA develops from 3 days to 11 months after trauma [2,3,4,5,11,39,40], and in some patients, after an unknown time since the trauma [38]. These patients demonstrate delayed clinical deterioration following the improvement of symptoms after trauma [2,3,4,11,40]. In patients with delayed ICH from a pseudoaneurysm in the MMA, CT angiography showed no EDH or ICH in contact with the dura mater [2,11,40]. We can expect these presentations given the mechanism of delayed ICH due to the pseudoaneurysm in the MMA. In delayed ICH from a pseudoaneurysm in the MMA, pressure from the pseudoaneurysm slowly thins the dura mater until it finally attaches to the inner layer of the dura mater rather than the outer layer. Consequently, the rupture of this pseudoaneurysm does not result in an EDH. Pseudoaneurysm adhesion to the brain cortex might promote the development of ICH or an SDH [5,6,39].

### 5.4. Importance of Preoperative Angiography

Pseudoaneurysms may be missed because cerebral angiography is seldom performed as a result of the introduction of CT and magnetic resonance imaging in patients after trauma. Higazi et al. reported the importance of angiography in the evaluation of pseudoaneurysms in the MMA after trauma [26]. Bozzetto-Ambrosi et al. reported a case of a 39-year-old man who underwent ICH removal [19]. The ICH developed after the patient fell down and hit his head on the floor [19]. Emergency craniotomy and ICH removal were performed without preoperative angiography. This patient showed a pseudoaneurysm in the posterior branch of the MMA as confirmed by postoperative angiography. Bozzetto-Ambrosi et al. treated the pseudoaneurysm with an endovascular approach [19]. If preoperative angiography had been performed, these authors could have removed the ICH and treated the pseudoaneurysm in one surgical operation. However, in the case of Bozzetto-Ambrosi et al., the operator thoroughly inspected the hematoma cavity and contents but found no particular abnormal tissue [19]. In this case, the bleeding pathology was located in the dura mater. A small craniotomy could not locate the pseudoaneurysm in the dura mater, thereby resulting in a second treatment.

**Figure 5:**
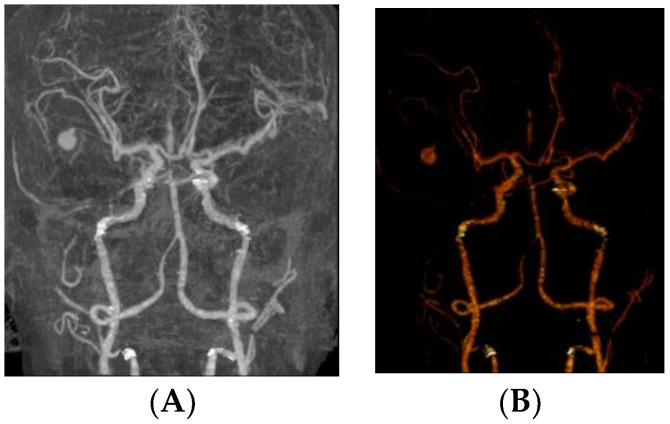
Computed tomography angiography before (**A**) and after reconstruction (**B**), showing a suspicious pseudoaneurysm without connection to an intracranial vessel.

Aoki et al. did not perform preoperative angiography before the removal of a recurrent acute SDH because of rapid progressive deterioration of the patient’s condition [4]. During the removal of the SDH, unexpectedly profuse bleeding developed from a pseudoaneurysm in the MMA. Aoki et al. suspected a pseudoaneurysm in the middle cerebral artery immediately after profuse bleeding occurred [4]. After failing to control the bleeding by temporary clipping of the M1 portion, Aoki et al. performed an extensive craniectomy [4]. Close inspection of the bleeding site showed that the lesion projected directly from the inner surface of the dura mater. After excising the pseudoaneurysm, hemostasis by coagulation of the MMA was easily achieved. Preoperative angiography provides useful information for the treatment of pseudoaneurysms in the MMA. Surgeons can expect the intraoperative rupture of pseudoaneurysms and avoid a second operation or later endovascular treatment. Reconstructive CT angiography can raise suspicions of a pseudoaneurysm. In CT angiography, pseudoaneurysms without a connection to an intracranial vessel have been observed (Figure 5). 

However, pseudoaneurysms are not always observed by CT angiography [2]. It is possible that CT angiography will miss a diagnosis of a pseudoaneurysm. We, therefore, recommend that CT angiography should be performed for patients with suspicion of a pseudoaneurysm in the MMA. If CT angiography does not identify a pseudoaneurysm, the clinician should perform cerebral angiography.

## 6. Operative Treatment

Pouyanne et al. reported one of the most dramatic presentations of delayed ICH of a pseudoaneurysm in the MMA in 1959 [5]. In that case, the patient fully recovered but then demonstrated a devastating temporal lobe hemorrhage one month later and showed severely impaired neurological status. A traumatic pseudoaneurysm in the MMA is not a benign lesion. The majority of studies report aneurysm growth or delayed hemorrhage with significant mortality. Therefore, clinicians should perform aggressive treatment for patients with ICH due to the rupture of the MMA after trauma. Although a few cases with spontaneous resolution of a pseudoaneurysm in the MMA have been reported [25,31], we recommend aggressive treatment, particularly if associated with ICH. For space-occupying ICH, patients should perform decompressive surgery. Whether endovascular treatment for MMA pseudoaneurysms is a preferred indication is unclear. 

### 6.1. Craniotomy

Wang et al. reported a case of an EDH only, without ICH, originating from a pseudoaneurysm in the MMA [29]. They described rapid bleeding from the skull base in an operation to remove the EDH; however, they could not locate the hemorrhage and could stop the bleeding only by a tenting suture during the EDH removal. In this operation, the operator did not suspect a pseudoaneurysm in the MMA and performed a craniotomy for the EDH removal. After the correct diagnosis of a pseudoaneurysm in the MMA by CT angiography, Wang et al. performed another extension craniotomy and identified an extradural pseudoaneurysm about 4 × 4 cm in diameter [29]. Correct craniotomy covering the skull base for the removal of a pseudoaneurysm in the MMA should be performed via the interpretation of CT angiography.

Aoki et al. reported extensive craniectomy, including the temporal base, after a failure to control bleeding by temporary clipping of the M1 portion during the removal of an SDH that originated from a pseudoaneurysm in the MMA [4]. The surgeon could successfully excise the pseudoaneurysm and control the bleeding. In operations for pseudoaneurysms in the MMA, surgeons should perform a wide craniotomy, including the proximal and distal MMAs of the pseudoaneurysm. 

### 6.2. Intraoperative Rupture from Pseudoaneurysm in MMA

In operations on acute ICH from pseudoaneurysms in the MMA, intraoperative rupture just after bone flap removal developed in two patients [6,7]. Moon et al. described a pseudoaneurysm attached to the outer surface of the dura mater accompanied by abrupt bleeding just after bone flap removal [6]. Kumar et al. also reported brisk extradural bleeding after bone flap removal [7]. 

In operations on delayed ICH from pseudoaneurysms in the MMA, intraoperative rupture from the pseudoaneurysm developed in two cases. One case developed active bleeding from the MMA after the dural opening [38]. In this case, the exact relationship between the dura mater and the pseudoaneurysm could not be identified. In another case, unexpected profuse bleeding from the pseudoaneurysm in the MMA developed just after the evacuation of the SDH [4]. Knowledge regarding the mechanism underlying delayed ICH development due to pseudoaneurysm suggests that the intraoperative rupture of a pseudoaneurysm can be expected during both the ICH removal and dural opening.

In our opinion, during operations for acute ICH removal, the intraoperative rupture of a pseudoaneurysm might occur just after bone flap removal because the pseudoaneurysm was attached to the outer surface of the dura mater. During surgery for delayed ICH removal, intraoperative bleeding from the pseudoaneurysm may occur after hematoma removal or dural opening because of the attachment of the pseudoaneurysm to the inner surface of the dura mater.

### 6.3. Treatment of Pseudoaneurysm

In cases of acute ICH from the rupture of a pseudoaneurysm in the MMA [6,7,8], we identified the detailed information for the treatment of pseudoaneurysms. In cases of delayed ICH [2,3,4,11,38,40], we obtained the operative information for the treatment of pseudoaneurysms. For operations on pseudoaneurysms, we found several characteristics of the pseudoaneurysms. In the operative field, Kumar et al. reported that the surgeon did not find a well-formed aneurysm wall [7]. In this case, cauterization of the MMA and occlusion of the foramen spinosum with bone wax were performed to control bleeding from the pseudoaneurysm. In acute ICH from a pseudoaneurysm in the MMA, a surgeon may not find a pseudoaneurysm in the operative field. Also, in reports on the surgical treatment for a pseudoaneurysm presenting as ICH, surgeons have excised the pseudoaneurysm and cauterized the MMA to control bleeding (Table 1 and Table 2). Moon et al. utilized the excision of a pseudoaneurysm and coagulated the MMA after several unsuccessful trials of cauterizations of the pseudoaneurysm for bleeding control [6]. Aoki et al. reported that after the excision of a pseudoaneurysm, hemostasis was easily controlled by coagulation of the MMA [4]. Sometimes, simple coagulation of the pseudoaneurysm can result in uncontrolled bleeding. We suggest that the excision of a pseudoaneurysm is the first step of the operation, followed by coagulation of the MMA.

## 7. Pathology of Pseudoaneurysm

**Figure 6:**
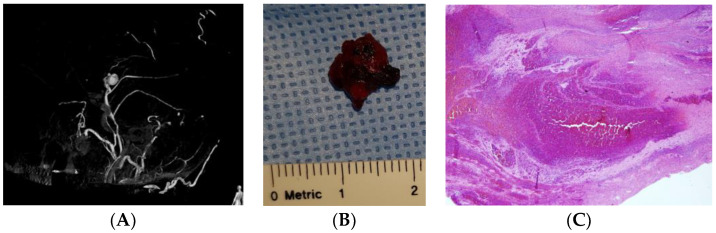
External carotid angiography shows a traumatic pseudoaneurysm located in the middle meningeal artery (**A**). Gross and (**B**) microscopic examination of a vascular lesion revealed a pseudoaneurysm (**C**). Hematoxylin and eosin stain (40×).

A partial rupture of the MMA after trauma can be repaired by clot formation, which contains intraluminal blood. Several researchers have reported pathological examination of the pseudoaneurysm in the MMA after trauma [4,11,23,26,29,30]. Microscopic examination of a pseudoaneurysm revealed a fibrotic capsule adjacent to a tear in the arterial wall [33]. Upon histological examination of a pseudoaneurysm presenting with acute ICH, the tissue had no normal vascular structure (Figure 6B,C). In delayed ICH presentation, histological examination of the aneurysmal sac demonstrated fibrous organization, organized hematoma, and deposits of hemosiderin [4,5].

## 8. Future Direction

More usage of CT angiography might result in the easy diagnosis of pseudoaneurysms in the MMA after trauma. Although the role of endovascular treatment for MMA pseudoaneurysms has not been well established in the neurosurgical field, endovascular embolization is a safe and effective method for eliminating re-bleeding in conditions of non-mandatory surgical decompression.

## 9. Brief Summary

In the development of a dura tear after trauma, the rupture of a pseudoaneurysm located in the epidural space produces a small EDH at the initial stage of the rupture and results in ICH in succession. In operations on acute ICH from a pseudoaneurysm in the MMA, intraoperative rupture just after bone flap removal may be developed. 

After the development of a pseudoaneurysm in the MMA due to trauma, pressure from the pseudoaneurysm slowly thins the dura mater until it finally attaches to the inner layer of the dura mater rather than the outer layer. In delayed ICH development due to pseudoaneurysm rupture, the intraoperative rupture of the pseudoaneurysm can be expected during both ICH removal and dural opening but not bone flap removal.

## 10. Conclusions

In cases of acute ICH due to the rupture of a pseudoaneurysm in the MMA, the pseudoaneurysm is in contact with the outer layer of the dura mater and usually ruptures during the removal of the bone flap. In cases of delayed ICH resulting from the rupture of a pseudoaneurysm in the MMA, the pseudoaneurysm is attached to the inner layer of the dura mater and may rupture upon dura opening or hematoma removal. Surgeons should perform extensive craniotomy surrounding a pseudoaneurysm and remain aware of the intraoperative rupture of pseudoaneurysms.

## Figures and Tables

**Table 1:** Clinical information of four patients with acute ICH due to rupture of pseudoaneurysm in MMA.

Reference	Age, Sex	Type of Trauma	Location of ICH	Operation Time from Trauma	Diagnostic Modality for PA before Operation	Location of PA	Intraoperative Rupture of PA	Treatment for PA
Kumar et al. [7]	53, Male	FD	Temporal lobe	Four days	CT angiography	No well-formed aneurysm	At bone flap removal	
Lim et al. [8]	70, Male	TA	Temporallobe	Two days	CT angiography	Inner surface of dura mater	Active bleeding from injured MMA	Parent vessel coagulation
Moon et al. [6]	60, Male	FD	Temporal lobe	Within 12 h	CT angiography and angiography	Outer surface of dura mater	At bone flap removal	Excision of PA
Wu et al. [2]	53, Male	TA	Frontal lobe	Within 24 h	CT angiography and angiography	NA	NA	Excision of PA

Abbreviations: ICH = intracerebral hemorrhage; MMA = middle meningeal artery; FD = fall down; TA = traffic accident; PA = pseudoaneurysm; CT = computed tomography; NA = not assessed.

**Table 2:** Clinical information of eight patients presented with delayed ICH due to rupture of pseudoaneurysm in MMA.

Reference	Age, Sex	Type of Trauma	Location of ICH	Operation Time from Trauma	Diagnostic Modality for PA before Operation	Location of PA	Intraoperative Rupture Time of PA	Treatment for PA
Aoki et al. [4]	75, M	TA	SDH	29 days	Non-enhanced CT	Fixed to the inner surface of DM	Just after evacuation of SDH	Excision of PA
Bruneau et al. [38]	64, F	UK	Frontal and temporal lobe	UK	Angiography	UK	Dural opening	Coagulation of PA
Kia-Noury et al. [39]	32, M	TA	Temporal lobe, SDH	7 days	Angiography	Fixed to the inner surface of DM	UK	
Kulanthaivelu et al. [40]	22, M	TA	Temporal lobe	11 days	Angiography	Fixed to the inner surface of DM	UK	Excision of PA
Montanari et al. [11]	66, M	FD	Frontal lobe	15 days	CT angiography	UK	UK	Coagulation of PA
Pouyanne et al. [5]	60, F	TA	Temporal lobe, SDH	30 days	Angiography	Fixed to the inner surface of DM	UK	
Singh et al. [3]	30, M	TA	Frontal lobe	11 months	Angiography	Fixed to the inner surface of DM	No bleeding	Coagulation of PA
Wu Et al. [2]	49, M	TA	Temporal lobe	16 days	Angiography	UK	No bleeding	Excision of PA

Abbreviations: ICH = intracerebral hemorrhage; MMA = middle meningeal artery; M = male; F = female; FD = fall down; TA = traffic accident; UK = unknown; SDH = subdural hemorrhage; PA = pseudoaneurysm; CT = computed tomography; DM = dura mater.

## Data Availability

The data supporting the reported results can be found in the references.
